# Cost-utility and cost-effectiveness of individual placement support and cognitive remediation in people with severe mental illness: Results from a randomized clinical trial

**DOI:** 10.1192/j.eurpsy.2020.111

**Published:** 2020-12-21

**Authors:** Thomas Nordahl Christensen, Marie Kruse, Lone Hellström, Lene Falgaard Eplov

**Affiliations:** 1 Copenhagen Research Center for Mental Health - CORE, Copenhagen, Denmark; 2 Danish Centre for Health Economics, University of Southern Denmark, Odense, Denmark

**Keywords:** Health economic analysis, individual placement and support, severe mental illness, supported employment

## Abstract

**Background:**

Administrators and policymakers are increasingly interested in individual placement and support (IPS) as a way of helping people with severe mental illness (SMI) obtain employment or education. It is thus important to investigate the cost-effectiveness to secure that resources are being used properly.

**Methods:**

In a randomized clinical trial, 720 people diagnosed with SMI were allocated into three groups; (a) IPS, (b) IPS supplemented with cognitive remediation a social skills training (IPSE), and (c) Service as usual (SAU). Health care costs, municipal social care costs, and labor market service costs were extracted from nationwide registers and combined with data on use of IPS services. Cost-utility and cost-effectiveness analyses were conducted with two primary outcomes: quality-adjusted life years (QALY) and hours in employment. Incremental cost-effectiveness ratios (ICER) were computed for both QALY, using participant’s responses to the EQ-5D questionnaire, and for hours in employment.

**Results:**

Both IPS and IPSE were less costly, and more effective than SAU. Overall, there was a statistically significant cost difference of €9,543 when comparing IPS with SAU and €7,288 when comparing IPSE with SAU. ICER’s did generally not render statistically significant results. However, there was a tendency toward the IPS and IPSE interventions being dominant, that is, cheaper with greater effect in health-related quality of life and hours in employment or education compared to usual care.

**Conclusion:**

Individual placement support with and without a supplement of cognitive remediation tends to be cost saving and more effective compared to SAU.

## Introduction

Although gainful employment repeatedly has been associated with better mental health and well-being, most people with severe mental illness (SMI) are unemployed [1–4]. This is an unfortunate situation, not only because employment has shown to contribute to recovery for the individual, but also because lost productivity generates significant costs to society besides the direct expenses of care and treatment [5].

International research has shown that the vocational rehabilitation intervention individual placement and support (IPS) is effective in helping people with SMI to obtain employment or education, and that training in cognitive and social functioning may increase the effects [6–8]. On this background, the effects of IPS, and IPS supplemented with cognitive remediation and work-related social skills training (IPSE) were investigated in a randomized, clinical trial (RCT) in Denmark during 2012–2018. The content of the interventions is thoroughly described in the trial protocol [9]. In short, the IPS intervention consisted of an individualized and rapid search for competitive employment based on the participants’ preferences. The intervention was integrated within the mental health services and the participants received time-unlimited support. The IPSE intervention consisted of IPS supplemented with 24 sessions of cognitive computer training aiming at improving basic cognitive functions such as attention, memory, and executive functioning. In addition, participants were taught cognitive coping and compensatory strategies. Moreover, the participants obtained training on work-related social skills focusing on how to disclose mental illness at the workplace, communication skills, decoding norms for social interaction, and conflict management.

The results of the trial showed that participants in the IPS group were more likely than those in the service as usual (SAU) group, to work competitively, or be enrolled in education, during the 18-month follow-up (59.9 vs 46.5%; SRD 0.134 [95% 0.009–0.257]). The difference between IPSE and SAU was 59.0 vs 46.5% (SRD 0.126; 95% CI 0.003–0.256). The IPS and IPSE participants also worked or studied more hours, and they were significantly more satisfied with the treatment received compared with the participants who received treatment as usual [10].

Despite IPS being established as an international evidence-based practice, only few cost-effectiveness studies of the intervention have been conducted [11–14]. The cost-effectiveness of IPS was investigated in six European cities, and IPS was found to produce better outcomes than alternative vocational services at lower cost overall to the health and social care systems [14]. However, the results varied along the labor market structure of the countries and did not attach monetary values to any observed improvements in health or quality of life. The Danish health care service is characterized by relatively easy access to psychiatric care and the labor market is characterized by good unemployment support, compared with many other countries [15]. These aspects may affect the cost-effectiveness of the IPS intervention compared with previous studies.

### Aims of the study

The overall aim of this study was to investigate the cost-utility in terms of quality-adjusted life years (QALY) and cost-effectiveness of IPS, in terms of hours in employment. The intention was that the results may inform policymakers, administrators in the job centers and health care planners in deciding future investments and implementation of vocational rehabilitation.

## Methods

Participants were recruited from community mental health services or early intervention teams (OPUS teams) in one of the three Danish cities; Copenhagen (including the municipality of Frederiksberg), Odense, or Silkeborg. Participants were eligible if they had a diagnose of schizophrenia, schizotypal, or delusional disorders (F20–F29) or bipolar disorder (F31), or recurrent depression (F33) according to the International Classification of Diseases and Related Health Problems—10th Revision (ICD 10). Participants had to be between 18 and 64 years old and they should express a clear aim of employment or education. Moreover, all participants should be assigned to early-intervention teams or community mental health services at one of the three included sites. To confirm that participants met the diagnostic criteria they were assessed by a trained and certified researcher using the diagnostic interview instrument The Schedules for Clinical Assessment in Neuropsychiatry version 2.184.

In total 720 individuals were randomly assigned into three arms; (a) IPS, *n* = 243, (b) IPSE, *n* = 238, and (c) SAU, *n* = 239. Participants allocated to SAU continued to receive counseling at the job centers and received treatment in early intervention teams (OPUS-teams) or community mental health treatment teams, in line with the two experimental groups.

All participants were assessed at baseline and 18-month follow-up in the period from 2012 to 2018 using researcher-administered semi-structured interviews, and self-reported questionnaires on outcome measures as social functioning, symptoms, self-esteem, and self-efficacy [10]. Health-related quality of life (HRQoL) was assessed using participants’ responses to the EuroQol five-dimensional questionnaire (EQ-5D) [16]. The self-administered instrument comprises five dimensions which are mobility, self-care, usual activities, pain/discomfort, and anxiety/depression. The participants self-rated their level of severity for each dimension using a three-level scale. (a) having no problems, (b) having some or moderate problems, and (c) being unable to do/having extreme problems. The validity and reliability of EQ-5D have been established across many conditions and populations and demonstrates good psychometric properties comparable to other generic measures, and it is one of the most frequently used measures in health-utility evaluations [17].

For each individual, the in- and out-patient costs in hospital care (both somatic and psychiatric care), primary health care costs, costs of pharmaceuticals, services provided by municipalities (labor market interventions and social service), and the IPS interventions costs were calculated accumulated within the follow-up period. The costs were assessed from a societal perspective meaning that costs outside the health care sector were included. Health care costs were obtained using the National Patient Register which is a key health register that covers somatic as well as psychiatric admissions, outpatient contacts, and emergency room contacts in all Danish hospitals [18]. Hospital costs were computed using nationally developed diagnosis-related groups tariffs [19]. Other health care costs, including costs in the primary sector and prescription medicine, were retrieved from the National Health Service Register [20] and the Pharmaceutical Database [21]. Costs of labor market interventions provided by the Danish job centers were obtained from register data in the Danish Agency for Labour Market and Recruitment. These interventions were primarily used by the SAU group and consisted of counseling at the job center, mentor support, or vocational rehabilitation interventions provided either by the job centers or private companies. Social services costs consisted of counseling, psychosocial initiatives, and personal assistance provided by the municipal social services. The costs of the IPS and IPSE interventions were calculated by using patient registration recorded by the IPS employment specialists or the psychologist who was responsible for the cognitive remediation groups. Only face-to-face contacts were included in the analyses, as was the case for the costs registered for the SAU group. Productivity gain was estimated by calculating hours in competitive employment multiplied by the average wage. If the productivity gain was positive, it counted as a negative cost and was therefore subtracted from total costs. All costs included in the analyses are described in more detail in Table 1. The average costs per participant were calculated in Euro (2016 price level), and the differences in costs between the intervention groups from baseline to follow-up were analyzed with *t*-tests. For the cost-effectiveness analyses a difference-in-difference approach was used by calculating the costs from baseline to 18 months follow-up deducted the costs from 18 months prior randomization.

**Table 1.** Cost components included in the cost-effectiveness analysis.

Costs	Definition	Source
Hospital costs	Inpatient, outpatient, and emergency room contacts in somatic and psychiatric hospitals, valued with DRG-tariffs.	The National Patient Register with DRG and outpatient tariffs [19,22].
Primary health care costs	Contacts to general practitioners, practicing specialists, and other health care professionals reimbursed (or partly reimbursed) by the Danish National Health Service, for example, dental care or psychological treatment. Costs are valued with national service tariffs.	The National Health Service Register [20].
Prescription pharmaceuticals	The full price (regardless of subsidies, etc.) of prescription drugs purchased in Danish pharmacies.	The Pharmaceutical Database [21].
Costs of labor market interventions	All interventions initiated by the municipal job centers: counseling, mentor support, or vocational rehabilitation interventions, primarily offered to the control group as part of SAU were valued at €20 per hour, mentor support in all groups was valued at €33 per hour and personal counseling in all groups was valued at €51 per hour. Education and on-the-job training were considered not to have additional costs.	Data obtained from the Danish Agency for Labour Market and Recruitment.
Costs of municipal social interventions	Social interventions, comprising counseling, psycho-social initiatives, and personal assistance and other means of non-monetary support.	Data obtained from Copenhagen municipality for those participants that lived in Copenhagen (70% of participants). Means per group were calculated and used throughout.
Intervention costs	Costs of the IPS intervention, valued at €1.33 per minute. The IPSE had an additional cost of €600 per patient.	Data obtained from the intervention, for participants from one site. The means for this site was used throughout.
Productivity	Productivity gain was estimated by calculating hours in competitive employment multiplied by the average wage. If the productivity gain was positive, it counted as a negative cost and was therefore subtracted from total costs.	Days in competitive employment are measured in the electronic income register from the Danish Agency for labor market and recruitment.

Abbreviations: DRG, Danish national diagnosis-related groups; IPS, individual placement and supports; SAU, service as usual.

QALY [23], and hours in employment were the effect measures in the present study. Traditionally, QALY are calculated by estimating the remaining life expectancy for a patient following a treatment or intervention multiplied with an HRQoL score (on a 0–1 scale). In the present study, however, we did not expect the IPS or IPSE interventions to have an impact on life expectancy beyond the intervention of 1.5 years. Thus, the difference between baseline and follow-up QALY measures only reflects HRQoL. EQ-5D scores were transformed into a single measure between 0 and 1 using the Danish preference weighting [16, 24]. The preference weights were calculated using a time trade-off survey among the general Danish population [24]. Discounting was deemed infeasible because of the uneven distribution of costs over the 18 months period (with most costs incurred at the beginning), and limited information about the distribution of health gain over the period.

### Cost-utility and cost-effectiveness

Cost utility was measured as the additional cost of gaining one additional QALY, or, in the present context, the additional cost of gaining one utility measure.

Incremental cost-effectiveness ratios (ICER) were computed as the difference between intervention groups and control group in costs, divided by the difference between groups in QALY gain from baseline to follow-up [25]:
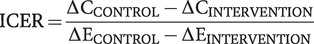


∆*C* denotes the difference in costs from 18 months before baseline, to 18 months after baseline. ∆*E* denotes the difference in QALY from baseline to follow-up. If the ICER was negative, it was interpreted as the treatment being dominant to the comparator, dominance meaning that the dominant treatment is more effective and costs less. The ICER’s were bootstrapped with 10,000 replications, and the 2.5 and 97.5 quantiles were interpreted as confidence limits. The bootstrapped analyses were visualized in a cost-effectiveness scatter plot [25, 26]. The plot presents the likelihood of getting a similar result if the experiment was repeated 10,000 times. The observations in the south-eastern quadrant of the plot represent cases where the intervention was both cheaper and better (dominant) in relation to QALY and thus worth implementing directly whereas the north-western quadrant represents cases where the intervention was more expensive and less effective (dominated) in which the intervention could simply be rejected. The north-eastern quadrant represents cases where the intervention was more expensive and better, and the south-western quadrant represents cases where the intervention was less expensive and less effective (Assess CE). In these cases, a more thorough health economic evaluation should be conducted before deciding if the intervention should be implemented. The primary analysis consists of complete cases, meaning that only participants who responded to EQ-5D at baseline and follow-up were included. However, as a sensitivity analysis, those missing at follow-up were included using multiple imputations (mi) with truncated regression in STATA. The regression analysis included EQ-5D at baseline, age, gender, and diagnosis as explanatory variables. Moreover, we conducted subgroups analyses on age (above or below median age), sex, diagnosis (mood disorders [F31/F33], and schizophrenia spectrum disorders [F2]), and education (primary/lower secondary education or higher educational degree).

The 10,000 bootstrap samples were used to generate a cost-effectiveness acceptability curve (CEAC) [25, 27]. The CEACs relate the ICER estimate to different monetary values of a QALY that decision-makers could be willing to pay. The CEAC was computed in a probabilistic sensitivity analysis where the probability of the treatment being cost-effective was evaluated at a societal threshold of €0 for willingness-to-pay for a QALY, up to a societal willingness to pay of €35,000. The latter limit is based on considerations from the Danish Health Technology Assessment guideline, according to which there is no official Danish threshold for willingness-to-pay for a QALY in Denmark but the €35,000 is often considered the upper limit [28].

Finally, cost-effectiveness was investigated in relation to hours in work and/or education in the follow-up period. The difference between groups in hours in work or education is presented with success rate difference derived from Wilcoxon’s U statistic, as in the original effectiveness study [10]. The ICER was calculated with the same methods as in the cost-utility analyses, and bootstrapped data were used to generate a cost-effectiveness plane where the two IPS groups combined are compared with SAU.

This study was conducted according to the Consolidated Health Economic Evaluation Reporting Standards statement. All analyses were conducted at the Statistics Denmark research server, where personal information about individuals is encrypted, thus ensuring compliance with data security regulations. SAS® v 9.4 was used for data management and STATA® MP v 15 was used for analysis. The authors assert that all procedures contributing to this work comply with the ethical standards of the relevant national and institutional committees on human experimentation and with the Helsinki Declaration of 1975, as revised in 2008.

## Results

Table 2 shows the baseline characteristics of the participants. The average age was 33 (SD 9.9) years, and 62% of the included participants were men. Most participants (77%) were diagnosed with a schizophrenia spectrum disorder, and the rest were diagnosed with bipolar affective disorder (12%) or recurrent depression (11%). Overall, the participants were relatively low educated with 39% having a primary or lower secondary education as the highest educational degree.

**Table 2.** Baseline characteristics of 720 participants in the trial.

	IPS (*N* = 243)	IPSE (*N* = 238)	SAU (*N* = 239)
Sex, *N* (%)			
Female	94 (38.7)	87 (36.6)	95 (39.8)
Male	149 (61.3)	151 (63.5)	144 (60.3)
Age, mean (SD)	33.3 (10.3)	33.0 (9.5)	32.8 (9.9)
Education, *N* (%)			
Master or equivalent	13 (5.4)	14 (5.9)	21 (8.8)
Bachelor or equivalent	28 (11.5)	22 (9.2)	28 (11.7)
Short-term tertiary education	43 (17.7)	53 (22.3)	44 (18.4)
Upper secondary education	61 (25.1)	57 (24.0)	57 (23.9)
Primary/lower secondary education	98 (40.3)	92 (38.7)	89 (37.2)
Diagnoses, *N* (%)			
Schizophrenia spectrum disorders (ICD10 Codes: F20-F29), *N* (%)	184 (75.7)	181 (76.1)	186 (77.8)
Bipolar disorder (ICD10 Codes: F31.0-F31.9), *N* (%)	32 (13.2)	30 (12.6)	25 (10.5)
Recurrent depression (Icd-10 F33.0-F33.9), *N* (%)	27 (11.1)	27 (11.3)	28 (11.7)
EQ-5D (SD)	0.71 (0.18)	0.69 (0.20)	0.70 (0.20)

Abbreviations: IPS, individual placement and support, IPSE, IPS + cognitive remediation and social skills training; SAU, service as usual.

There was no clinically relevant difference between the three groups in any baseline measures. 64% (*N* = 462) of the participants answered the EQ-5D questionnaire at baseline and follow-up and could be included in the complete case analyses. There was no significant difference in the dropout rates between the three groups and no significant difference in baseline EQ-5D score between those who answered EQ-5D at follow-up and those who did not.

Table 3 shows the total costs during the 18-month follow-up period. For participants in IPS, the costs of psychiatric hospital care were €3,730 lower per person, compared to the SAU-group, and the IPSE group had €4,545 lower costs in psychiatric hospital care compared to the participants who received SAU. The IPS and IPSE participants also had statistically lower costs of labor market interventions provided by the job centers, compared with SAU. In addition, IPS participants earned an average of €1,792 and IPSE €756 more than the control group, meaning that the production gains were higher in the two IPS groups. Overall, there was a statistically significant cost difference of €9,543 when comparing IPS with SAU and €7,288 when comparing IPSE with SAU (Table 3).

**Table 3.** Costs and QALY’s during the 18 months after randomization, EURO.

Costs	IPS costs	SAU costs	Probability of equality of means IPS vs SAU	IPSE costs	Probability of equality of means IPSE vs SAU
Somatic hospital	1,447	1,573	0.7293	1,260	0.3209
Prescription pharmaceuticals	1,438	1,377	0.7877	943	0.0237
Primary health care	286	286	0.9972	271	0.6120
Mental health hospital care	14,549	18,279	0.0961	13,743	0.0426
Labor market interventions	403	3,395	<0.0001	415	<0.0001
Municipal social interventions	1,759	3,636	N/A	3,121	N/A
intervention costs	914	0	N/A	2,543	N/A
Productivity gain (subtracted from total costs)	−7,214	−5,422	0.2052	−6,458	0.4351
Total costs	13,582	23,125	0.0010	15,837	0.0106
QALY gain	0.0329	0.0074	0.2960	0.0702	0.0146

Abbreviations: IPS, individual placement and support; IPSE, IPS + cognitive remediation and social skills training; QALY, quality adjusted life years, SAU, service as usual.

In Table 4, QALY gains and the resulting ICER are shown, based on the complete case analysis, that is, where patients with missing QALY information were excluded from the analysis. For all groups, there were improvements in QALY. The gains in the experimental groups were greater than in the control group. The largest gain was seen for IPSE, which was significantly greater than the gain seen in the control group (the difference was 0.063 [95% CI 0.012–0.113]). In both IPS groups, the ICER was dominant, that is, cheaper with greater effect, but these results were not statistically significant. When comparing the two intervention groups the IPSE group had a higher gain in HRQoL, but at an extra cost, when compared with IPS.

**Table 4.** Cost-effectiveness results, complete case analysis, costs in EURO’s.

	Cost development, € (95% CI)	QALY gained (95% CI)	ICER, € per QALY gained (95% CI obtained by bootstrapping)
IPSE vs SAU *N* = 295			
IPSE (*N* = 145)	**−8,951** (−14,107; −3,794)	**0.070** (0.033; 0.107)	Dominant (−393,892; 57,516)
SAU (*N* = 150)	−4,687 (−9,813; 440)	0.007 (−0.027; 0.042)	
Difference	−4,264 (−11,506; 2,978)	**0.063** (0.012; 0.113)	
IPS vs SAU *n* = 317			
IPS (*N* = 167)	**−10,219** (−15,241; −5,198)	0.033 (−0.000; 0.066)	Dominant (−2.08e+7; 229,165)
SAU (*N* = 150)	−4,687 (−9,813; 440)	0.007 (−0.027; 0.042)	
Difference	−5,533 (−12,694; 1,628)	0.025 (−0.022; 0.073)	
IPSE vs IPS *n* = 312			
IPSE (*N* = 145)	**−8,951** (−14,107; −3,794)	**0.070** (0.033; 0.107)	33.953 (−518,284; 85,311)
IPS (*N* = 167)	**−10,219** (−15,241; −5,198)	0.033 (−0.000; 0.066)	
Difference	1,269 (−5,923; 8,461)	0.037 (−0.012; 0.087)	

Note: Figures in bold are statistically significant at 5% level.

Abbreviations: CI, confidence interval; ICER, incremental cost effectiveness; IPS, individual placement and support; IPSE, IPS + cognitive remediation and social skills training; QALY, quality adjusted life years; SAU, service as usual.

The IPS and IPSE groups remained dominant compared to SAU when using imputed data. However, the difference in HRQoL between the groups was reduced while the cost difference increased (online supplementary table 1). In subgroup analyses on age, sex, diagnosis, and education, IPS and IPSE also remained dominant to SAU. However, it seems that the cost difference was driven by those with a primary/lower secondary education, while the difference in HRQoL was driven by those with a higher educational degree. For a full overview, all subgroup analyses are available in the online supplementary material (Tables 1–8).

**Figure 1.**
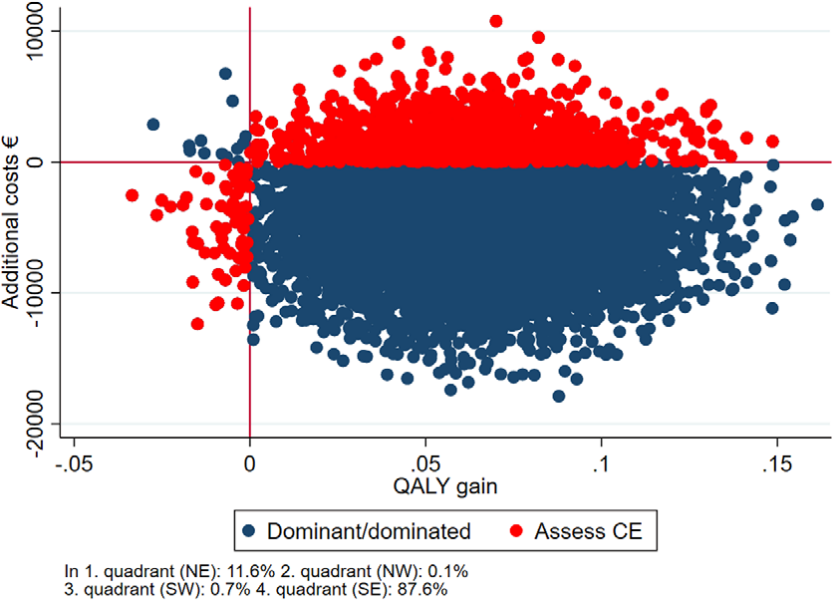
Cost-effectiveness plane IPSE vs SAU, complete case analysis.

**Figure 2.**
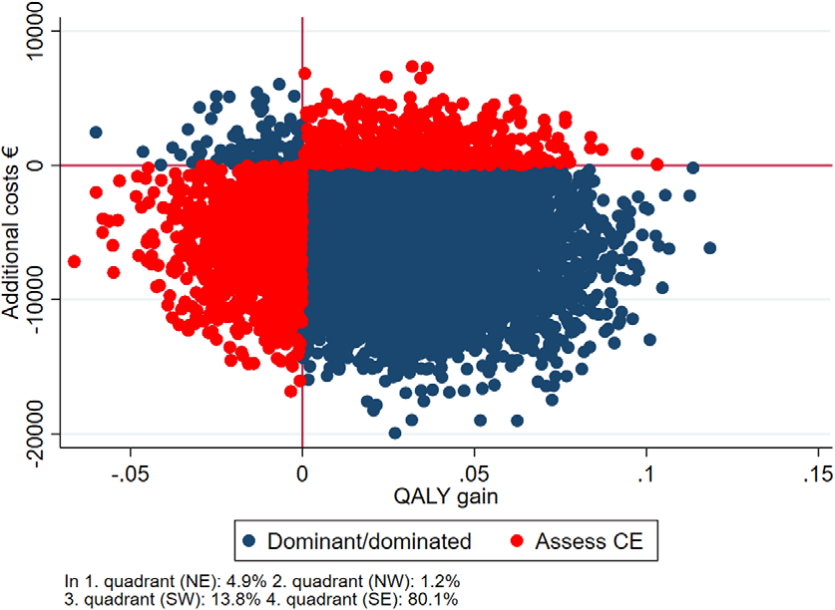
Cost-effectiveness plane, IPS vs SAU, complete case analysis.

**Figure 3.**
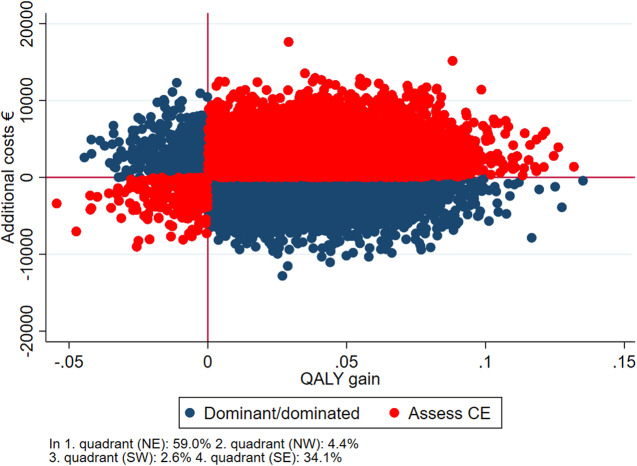
Cost-effectiveness plane, IPSE vs IPS, complete case analysis.

Figures 1–3 reflect the cost-effectiveness (ICER) results presented in Table 4. IPS and IPSE appear to be dominant compared with SAU. When comparing IPSE with SAU 88% of the scattered dots of ICERs were located in the SE quadrant, that is, better and cheaper, while this was the case for 80% of the dots when IPS was compared with SAU. Overall, IPS and IPSE were superior to SAU in terms of higher HRQoL and lower costs, albeit not statistically significant.

**Figure 4.**
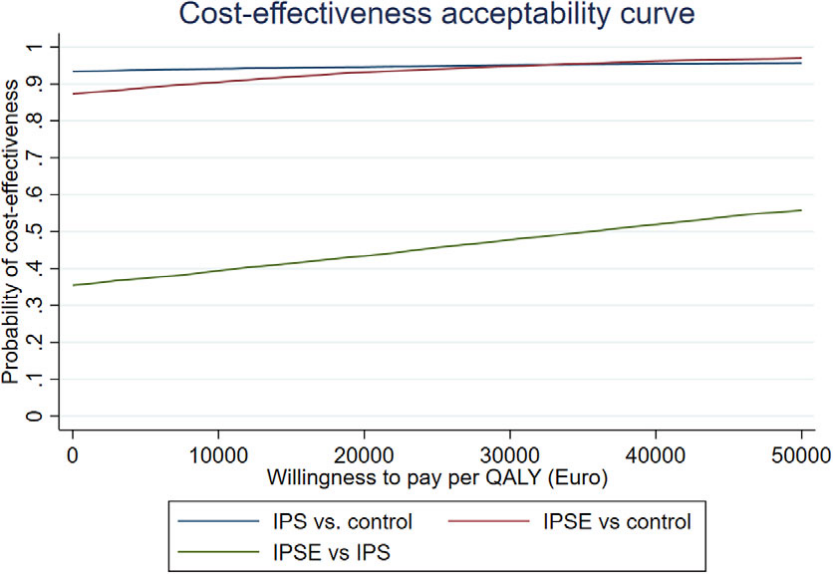
Cost-effectiveness acceptability curve.

A probabilistic sensitivity analysis was conducted where the probability of IPS or IPSE being cost-effective was evaluated at different societal thresholds for willingness to pay for a QALY. Based on the lack of variation in this analysis, the uncertainty of the estimates was considered minor important. With a societal threshold of €0 for willingness-to-pay for a QALY, corresponding to the case where society is unwilling to pay for a QALY gain, there is a probability of 88.3% of IPSE being cost-effective because IPSE in most cases is dominant, cheaper, and better. At a societal willingness to pay of €35,000, the probability is more than 95%. For IPS vs SAU, the probability of cost-effectiveness at €35,000 is 95.6%. When comparing IPSE and IPS, the probability of cost-effectiveness only exceeds 50% at a societal threshold of €35,000 (Figure 4).

**Figure 5.**
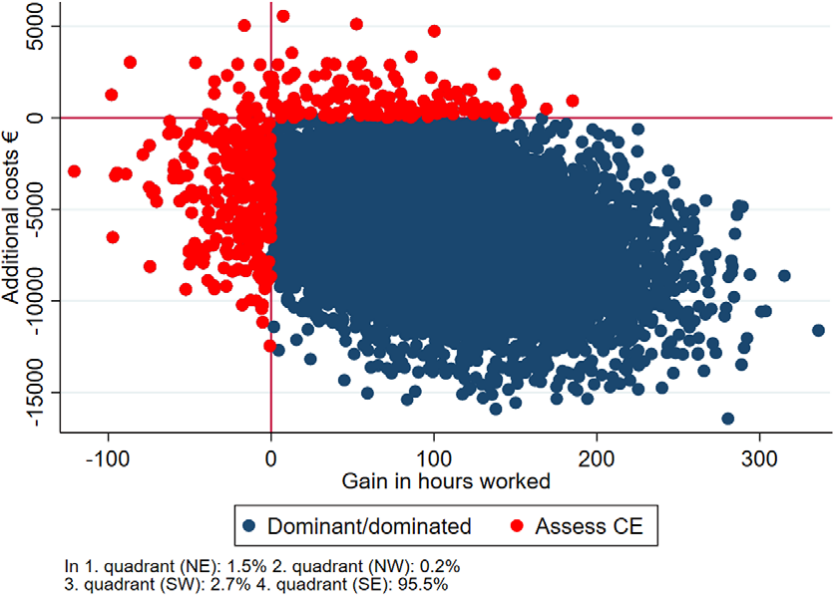
Cost-effectiveness plane of hours in work or education vs costs, between IPS + IPSE vs SAU.

Table 5 and Figure 5 reflect the cost-effectiveness in terms of the number of hours in work or education during the 18-month follow-up period. The two IPS groups worked and studied significantly more hours when compared to SAU. (448 vs 341 h, *p* = 0.002) and at an overall lower cost (€−6,214). The ICER shows that IPS and IPSE are dominant to SAU where 95.5% of the scattered dots of ICERs were located in the SE quadrant, that is, better and cheaper.

**Table 5.** Cost-effectiveness results, complete case analysis, costs in EURO’s, and hours obtained in employment and education.

	Cost development, € (95% CI)	Hours in employment or education (95% CI)	ICER, € per hour gained (95% CI)
IPS + IPSE vs SAU (*N* = 521)			
IPS + IPSE (*N* = 356)	**−10,284** (−13,772; −6.812)	448 (375; 520)	Dominant (−486–5)
SAU (*n* = 165)	−4,079 (−8,702; 545)	341 (254; 427)	
Difference	**−6,214** (−12,176; −251)	**107** SRD = 0.138 (0.009; 0.263; *p* = 0.002)^a^	

Note: Figures in bold are statistically significant at 5% level.

Abbreviations: CI, confidence interval; IPS, individual placement and support; IPSE, IPS + cognitive remediation and social skills training; QALY, quality adjusted life years; SAU, service as usual; SDR, success rate difference.

a Success rate difference derived from Wilcoxon’s U statistic.

## Discussion

IPS and IPS supplemented with cognitive remediation were less costly than SAU, with €9,543 lower costs (IPS vs SAU) and €7,288 lower costs (IPSE vs SAU). Additionally, there was a slight improvement in QALY after 18 months in the two IPS groups. However, this gain was only statistically significant among the IPSE participants when compared with SAU. The incremental cost-effectiveness ratio indicated that IPS and IPSE were dominant, for example, both better (measured in QALY) and cheaper compared to SAU, but these results were not statistically significant. However, the results appear robust when data were bootstrapped and visually presented in a scatter plot. In addition, the two IPS groups were cost-effective compared to hours in work or education. Participants in both IPS groups worked or studied more hours and had lower costs compared with SAU.

The lower costs in the IPS and IPSE groups reflected in part the positive effects of IPS on labor market affiliation but most of the difference was related to consistently lower health care costs and municipal costs in both experimental groups. The reasons for the reduced health care costs in IPS and IPSE are likely multifaceted. One explanation, and a commonly used argument, is that participation in IPS improves participants’ social functioning which results in less need for services and lower costs for mental health care [12]. Another explanation may be that work in itself mediates symptom reduction and enhance self-esteem, which reduces the need for psychiatric treatment [11, 29]. As reported earlier in the effectiveness study there were no statistically significant differences in social functioning or any psychiatric symptoms between groups which makes the second explanation more reasonable [10]. This is also supported by results from a correlation study on the RCT, where those who obtained employment or education had higher self-esteem and functioning and less psychiatric symptoms compared to those who did not. Furthermore, the difference in lower costs between the IPS groups and SAU was mainly driven by outpatient contacts and not hospitalization [10]. This could be explained by the high integration of IPS within local mental health services in the present study. During the trial, the psychiatric case managers informed that after IPS was implemented they used less time on social work and collaboration with the staff at the job centers. Hence, the patients may have had less need for contacts with the psychiatric case managers because counseling in social benefits and support for finding and retaining employment or education were delegated to the IPS employment specialists.

Previous IPS cost-effectiveness studies have also demonstrated lower health care costs among IPS participants compared with control groups, but the differences have been less pronounced than the findings in the present study. In the Supported Work and Needs trial by Heslin et al. [30] it was found that IPS participants had fewer days in hospital and outpatient care compared with SAU participants giving a cost difference of £2,361 in favor of the IPS intervention, but this was not statistically significant. In a cost-effectiveness study of IPS in six European cities by Knapp et al., the IPS group had significantly lower cost in inpatient services than participants receiving SAU in the first 12 months of the study [14]. However, the difference diminished over the subsequent 12 months, and there were no differences between groups related to outpatient care. In a study by Dixon et al., no statistically significant differences in mental health costs were found between IPS and control group participants [12]. Compared to previous trials the cost difference in mental health care estimated in the present study of €3,730 and €4,545 between IPS and IPSE vs SAU is considerable.

To the best of our knowledge, no other IPS studies for people with SMI include QALY as an effect measure, and therefore no results can frame the findings of the present study. However, a Swedish RCT investigating the effects of supported employment adapted for people with affective disorders found an insignificant QALY gain of 0.046 (95% CI −0.05 to 0.13) in the supported employment group, and 0.056 (95% CI −0.06 to 0.17) in the group who received traditional vocational rehabilitation [31]. In the present study, a small gain in QALY was seen in all three groups, but mostly in the IPSE group with a statistically significant gain of 0.07, and a significant difference of 0.063 when compared with SAU. These points toward improved mental health among the IPSE participants, which most likely have been generated by the additional provision of cognitive remediation and social skills training in this group. However, there were no differences between IPSE and SAU in any other non-vocational outcomes, such as cognitive function, level of depression, or social functioning in the original effectiveness study. The increased HRQoL in the IPSE group may then be explained by the higher rates of employment and education, rather than the cognitive remediation, which again could contribute to explaining the lower mental health care costs. However, it could also be that the additional training in this group was too time-consuming and therefore resulted in fewer outpatient psychiatric contacts.

A major strength of the present health economic analysis was the access to population-based register-based data on both health care costs and costs in the municipalities and national Danish employment agencies. There are also a few limitations. Most importantly, we had limited knowledge about treatment received outside of the public sector. Services such as psychotherapy and job coaching may have been purchased in the private sector. Another limitation is the scarce information about municipal services. We only had access to information from Copenhagen municipality and therefore had to apply group averages computed on Copenhagen data on the entire population, hence not capturing the variance of these costs.

In conclusion, this study presents a strong case for implementation of IPS and IPSE in a population of individuals with schizophrenia, bipolar, and other affective disorders in Denmark. Apart from supporting more participants to education and competitive employment, the costs of the two IPS groups were lower, and the HRQoL was higher when comparing with SAU. However, these positive effects are not guaranteed in future implementation. Variations in financing and contracting and change in the labor market policies, as well as the ability of providers to implement the service with high fidelity, are all likely to shape the cost and effectiveness of IPS.

## Data Availability

Data supporting the findings of this study are not publicly available due to legal restrictions from the Danish data protection agency and the European data protection regulation. Data are hosted by Statistics Denmark and only the authors of this study are allowed access.

## Supplementary material

For supplementary material accompanying this paper visit https://doi.org/10.1192/j.eurpsy.2020.111. click here to view supplementary material
