# Supplementary material for: Cost-utility and cost-effectiveness of individual placement support and cognitive remediation in people with severe mental illness: Results from a randomized clinical trial
Source: Eur Psychiatry. 2020 Dec 21;64(1):e3. doi: 10.1192/j.eurpsy.2020.111 (PMC8057485; doi:10.1192/j.eurpsy.2020.111)
Supplement: Supplementary file 1 [file S092493382000111Xsup001.docx]

Table 1 Cost-effectiveness results with imputed data, costs in EURO’s

|  | Cost development, €  (95% CI)) | QALY’s gained (95% CI) | ICER, € per QALY gained  (95 % CI obtained by bootstrapping) |
| --- | --- | --- | --- |
| IPS vs SAU N=450 | | | |
| ips (N=230) | -10,604 (-220,869; 78,273) | -.032 (-.631; .803) | Dominant (-1.46e+08; 99,234) |
| sau (N=220) | -1,983 (-134,269; 126,684) | -.048 (-.672; 1.022) |  |
| Difference | -8,621 (-14,811; -2,360) | .0172 (-.026; .061) |  |
| IPSE vs SAU n=436 | | | |
| ipsE (N=216) | -6,291 (-159,324; 197,262) | -.013 (-.591; .851) | Dominant (-2,399,080; 280,966) |
| sau (N=220) | -1,983 (-134,269; 126,684) | -.048 (-.672; 1.022) |  |
| Difference | -4,308 (-10,208; 1,800) | .036 (-.011; .086) |  |

Table 2 Cost-effectiveness results among participants with primary/lower secondary education as highest educational degree

|  | Cost development, €  (95% CI)) | QALY’s gained (95% CI) | ICER, € per QALY gained  (95 % CI obtained by bootstrapping) |
| --- | --- | --- | --- |
| IPS+ipse (N= 118) | -11,227 (-194,042; 107,145) | .037 (-.591; 851) | Dominant (-6.40e+08; -6.40e+08) |
| sau (N= 49) | 865 (-134,269; 126,684) | .037 (-.596; 367) |  |
| Difference | -12,092 (-23,713; -389) | .0001 (-.065; 070) |  |

Table 3 Cost-effectiveness among participants with a higher educational degree than primary/lower secondary education

|  | Cost development, €  (95% CI)) | QALY’s gained (95% CI) | ICER, € per QALY gained  (95 % CI obtained by bootstrapping) |
| --- | --- | --- | --- |
| IPS+ipse (N=194) | -8,657 (-113,097; 78,273) | .058 (-.591; .84) | Dominant (-204,522; 148,560) |
| sau (N=101) | -7,380 (-92,555; 60,588) | -.007 (-.531; 1.022) |  |
| Difference | -1,278 (-8,222; 5,913) | .065 (.011; .118) |  |

Table 4 Cost-effectiveness among male participants

|  | Cost development, €  (95% CI)) | QALY’s gained (95% CI) | ICER, € per QALY gained  (95 % CI obtained by bootstrapping) |
| --- | --- | --- | --- |
| IPS+ipse (N=189) | -8,604 (-194,041; 107,144) | .060 (-.482;.851) | Dominant (-1,008,574; 55,034) |
| sau (N= 83) | -1,538 (-92,554; 75,980) | .003 (-.596; 1.022) |  |
| Difference | -7,065 (-14,390; 380) | .057 (-.003; .115) |  |

Table 5 Cost-effectiveness among female participants

|  | Cost development, €  (95% CI)) | QALY’s gained (95% CI) | ICER, € per QALY gained  (95 % CI obtained by bootstrapping) |
| --- | --- | --- | --- |
| IPS+ipse (N=123) | -11,205 (-159,324; 38,788) | .035 (-.591; .799) | Dominant (-1.55e+07; 327,224) |
| sau (N=67) | -8,586 (-134,269; 126,684) | .012 (-.474; .531) |  |
| Difference | -2,619 (-12,860; 7,643) | .022 (-.039; .083) |  |

Table 6 Cost-effectiveness among participants below 32 years

|  | Cost development, €  (95% CI)) | QALY’s gained (95% CI) | ICER, € per QALY gained  (95 % CI obtained by bootstrapping) |
| --- | --- | --- | --- |
| IPS+ipse (N=161) | -5,847 (-194,041; 107,144) | .071 (-.591; .851) | Dominant (-1,103,038; 242,391) |
| sau (N=68) | -1,019 (-134,269; 126,684) | .016 (-.596; .529) |  |
| Difference | -4,828 (-14,960; 5,158) | .055 (-.007; .117) |  |

Table 7 Cost-effectiveness among participants above 31 years

|  | Cost development, €  (95% CI)) | QALY’s gained (95% CI) | ICER, € per QALY gained  (95 % CI obtained by bootstrapping) |
| --- | --- | --- | --- |
| IPS+ipse (N=151) | -13,662 (-159,324; 57,965) | .028 (-.591; .799) | Dominant (-1.53e+07; 169,365) |
| sau (N=82) | -7,727 (-87,522; 52,223) | -.00 (-.531; 1) |  |
| Difference | -5,934 (-13,690; 1,892) | .028 (-.030; .084) |  |

Table 8 Cost-effectiveness among participants diagnosed with recurrent depression or bipolar disorder

|  | Cost development, €  (95% CI)) | QALY’s gained (95% CI) | ICER, € per QALY gained  (95 % CI obtained by bootstrapping) |
| --- | --- | --- | --- |
| IPS+ipse (N=70) | -11,711 (-143,359; 27,828) | .058 (-.546; .529) | Dominant (-3,800,205; 333,732) |
| sau (N=37) | -7,337 ( -84,267; 52,223) | -.011; (-.531; .367) |  |
| Difference | -5,209 (-12,432; 2,390) | .035 (-.015; .084) |  |

Table 9 Cost-effectiveness among participants diagnosed with schizophrenia spectrum disorder

|  | Cost development, €  (95% CI)) | QALY’s gained (95% CI) | ICER, € per QALY gained  (95 % CI obtained by bootstrapping) |
| --- | --- | --- | --- |
| IPS+ipse (N=242) | -9,027 (-194,041; 107,144) | .048 (-.591; .851) | Dominant (-879,594; 225,425) |
| sau (N=113) | -3,818 (-134,269; 126,684) | .013 (-.596; 1.0) |  |
| Difference | -4,374 (-15,389; 6,588) | .069 (-.012; .151) |  |
